# Histone posttranslational modifications and cell fate determination: lens induction requires the lysine acetyltransferases CBP and p300

**DOI:** 10.1093/nar/gkt824

**Published:** 2013-09-12

**Authors:** Louise Wolf, Wilbur Harrison, Jie Huang, Qing Xie, Ningna Xiao, Jian Sun, Lingkun Kong, Salil A. Lachke, Murali R. Kuracha, Venkatesh Govindarajan, Paul K. Brindle, Ruth Ashery-Padan, David C. Beebe, Paul A. Overbeek, Ales Cvekl

**Affiliations:** ^1^Department of Ophthalmology and Visual Sciences, Albert Einstein College of Medicine, Bronx, NY10461, USA, ^2^Department of Genetics, Albert Einstein College of Medicine, Bronx, NY10461, USA, ^3^Department of Molecular and Cellular Biology, Baylor College of Medicine, Houston, TX 77030, USA, ^4^Departments of Ophthalmology and Visual Sciences, Washington University Saint Louis, Saint Louis, MO 63110, USA, ^5^Department of Biological Sciences, University of Delaware, Newark, DE 19716, USA, ^6^Center for Bioinformatics and Computational Biology, University of Delaware, Newark, DE 19716, USA, ^7^Department of Surgery, Creighton University, Omaha, NE 68178, USA, ^8^Department of Biochemistry, St. Jude Children’s Research Hospital, Memphis, TN 38105, USA and ^9^Department of Human Molecular Genetics and Biochemistry, Sackler School of Medicine and Sagol School of Neuroscience, Tel Aviv University, Israel 69978

## Abstract

Lens induction is a classical embryologic model to study cell fate determination. It has been proposed earlier that specific changes in core histone modifications accompany the process of cell fate specification and determination. The lysine acetyltransferases CBP and p300 function as principal enzymes that modify core histones to facilitate specific gene expression. Herein, we performed conditional inactivation of both CBP and p300 in the ectodermal cells that give rise to the lens placode. Inactivation of both CBP and p300 resulted in the dramatic discontinuation of all aspects of lens specification and organogenesis, resulting in aphakia. The CBP/p300^−/−^ ectodermal cells are viable and not prone to apoptosis. These cells showed reduced expression of Six3 and Sox2, while expression of Pax6 was not upregulated, indicating discontinuation of lens induction. Consequently, expression of αB- and αA-crystallins was not initiated. Mutant ectoderm exhibited markedly reduced levels of histone H3 K18 and K27 acetylation, subtly increased H3 K27me3 and unaltered overall levels of H3 K9ac and H3 K4me3. Our data demonstrate that CBP and p300 are required to establish lens cell-type identity during lens induction, and suggest that posttranslational histone modifications are integral to normal cell fate determination in the mammalian lens.

## INTRODUCTION

Cellular differentiation and organogenesis depend on precise temporal and spatial control of gene expression. During embryonic development, individual cell types are specified through the selective activation and repression of groups of genes. Genomic DNA in the nucleus is packaged as a nucleoprotein polymer termed chromatin. The structure of the chromatin plays an important role in regulating expression levels for individual genes. Eukaryotic cells alter chromatin structure primarily via posttranslational modifications (PTMs) of histones, and ATP-dependent remodeling of nucleosomes.

CBP (Crebbp, KAT3A) and p300 (Ep300, KAT3B) are two members of the KAT3 subfamily of histone K-acetyltransferases (HATs). The histone acetyltransferase family is composed of 20 distinct enzymes with various substrate specificities (1). Their major acetylation targets include histone H2A at lysine 5 (K5), histone H2B at K12, 15 and 20 and histone H3 at K14, 18 and 27. On histone H4, CBP and p300 preferentially modify K12 and K8 residues, respectively (2). Global H3 K18ac and K27ac levels are dramatically reduced in CBP and p300 double null mouse fibroblasts (3,4), suggesting that these HATs are chiefly responsible for these PTMs. H3 K27 acetylation is strongly correlated with active enhancers and promoters (5,6).

Gene targeting studies of p300 and CBP revealed that CBP and p300 are not fully redundant. Germline inactivation of p300 resulted in multiple defects in organogenesis, including defects in neural tube and heart formation, attributed to impaired proliferation of the p300 null cells (7). CBP null embryos developed abnormal blood vessels and massive hemorrhage that caused death at E10.5 (8). In addition, abnormalities were found in hematopoiesis, neural tube closure and embryonic growth. Loss of one copy of CBP was sufficient to induce defects in hematopoietic differentiation (9). The studies in mice are consistent with human genetic studies that identified CBP, and, less frequently, p300, as genes mutated in Rubinstein–Taybi syndrome (10). Patients typically have mental retardation, heart disease and malformed thumbs and toes, and often exhibit ocular defects including cataracts and glaucoma (10,11). To study CBP and p300 functions in specific cell types, conditional gene targeting was previously performed in T-cell lymphocytes (12), in renin-positive juxtaglomelular kidney cells (13) and in photoreceptors (14). These studies found that CBP and p300 are essential for T-cell formation (12), necessary for maintenance of renin cell identity (13) and required for photoreceptor-specific gene expression (14).

**Figure 1.**
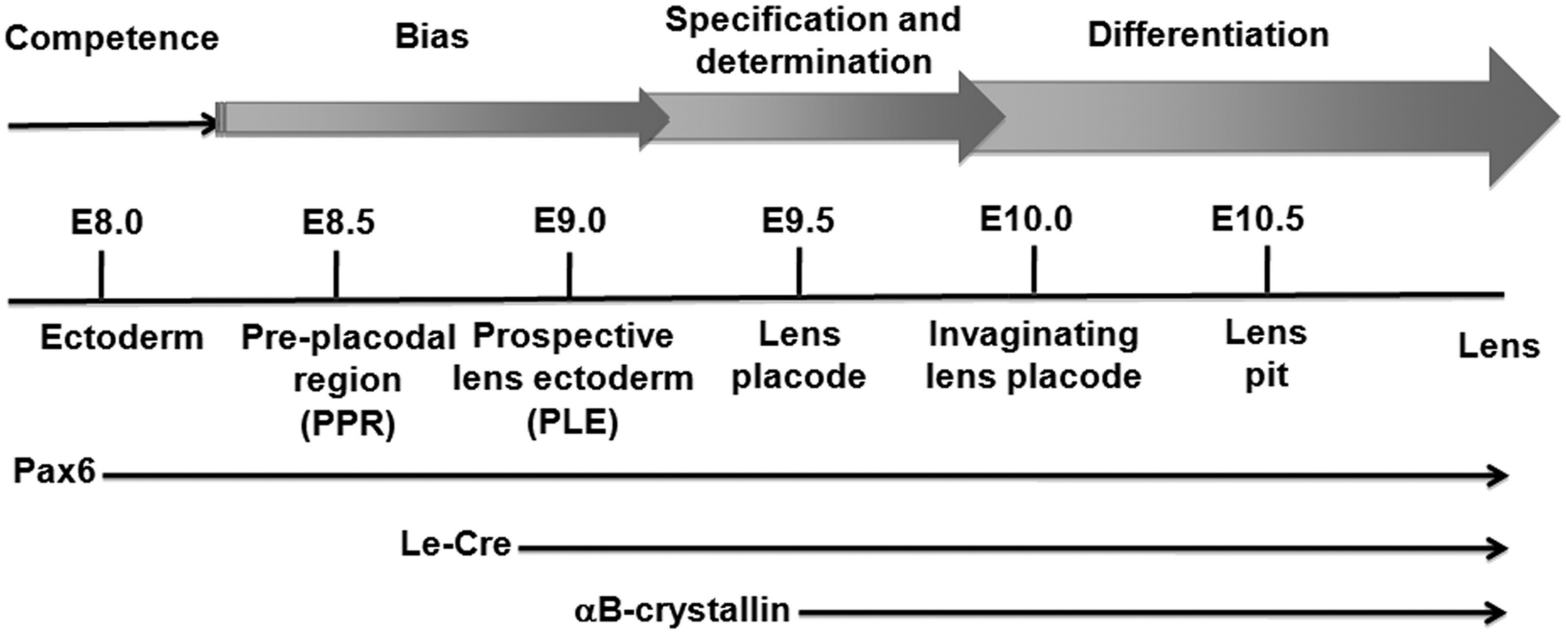
Schematic overview of lens induction in the mouse embryo. Top: Earlier experiments have defined four stages of lens development termed competence, bias, specification and determination, and differentiation (15–21). Middle: Stages of lens morphogenesis are shown at 0.5 day intervals. Lower: Horizontal arrows indicate the timing of expression of the endogenous Pax6 gene, the Le-Cre transgene and the endogenous αB-crystallin gene.

Lens induction provides an advantageous model system to study both cell fate determination and tissue morphogenesis (15–17). The phenomenon of embryonic induction involves commitment to, or specification of, a new cell fate resulting from the directed exchange of signals between the ‘inducing’ and ‘induced’ cells. In the case of the lens, induction is the final step in a multistage process (see Figure 1) (15,18,19). At the outset, lens bias and competence are transitional properties acquired by ectodermal cells located in proximity to the anterior neural plate within the preplacodal region (Figure 1, E8.0–E8.5) (20). Preplacodal cells give rise to the entire spectrum of cranial placodes, including the adenohypophyseal, olfactory, lens and otic placodes (21,22). The lens progenitor cells become positioned in the region of the head where the lens placodes will be induced (Figure 1, E8.5–E9.0) (23). Concurrently, mesoderm- and endoderm-derived tissues, sources of early inductive signals, retract from the head region, and the neuroectoderm/prospective optic vesicles extend bilaterally toward the ectoderm. This results in the lens-specification stage (Figure 1). At this stage, the presumptive lens cells are still developmentally flexible (24–26). Lens cell determination and lens morphogenesis are triggered when the bilateral optic vesicles come into contact with the overlying lens progenitor cells (15). The specified cells elongate and form the lens placodes (Figure 1, E9.5). Each lens placode subsequently invaginates to form a lens pit (Figure 1, E10.0–E10.5). This is accompanied by invagination of each optic vesicle to form an optic cup. Each lens pit seals around the edges and detaches from the surface ectoderm to form the lens vesicle. The posterior cells of the lens vesicle exit from the cell cycle and differentiate into the highly elongated primary lens fiber cells (27).

The molecular events that define the cellular commitment to a new differentiation program during lens induction, and during induction in general, are not yet fully understood. At the level of chromatin, it has been proposed that the nuclear organization of chromosomes as well as the local organization of individual genes may be altered, and, may thereby govern the formation of individual differentiated cell types (28,29). Alternatively, or concurrently, a wide range of chromatin modifications may be used to establish novel patterns of gene expression. Core histone PTMs, including acetylation/deacetylation and methylation/demethylation, are postulated to play major roles in chromatin organization and in gene regulation, and thus may play critical roles in cell fate determination (30,31). It has not yet been determined which of these alterations are central to the inductive process in any cell type.

Chromatin remodelers are proposed to partner with specific DNA binding transcription factors that recruit them to individual regulatory domains, including promoters and enhancers. Genetic studies in mice have demonstrated that a group of three transcription factors (Pax6, Six3 and Sox2) are required for lens progenitor cell formation (32,33). The commonly shared chromatin remodelers that are known to interact with these factors are CBP and p300 (34). In this study, we aimed to analyze the function of these remodelers in lens development. Our data demonstrate that CBP and p300 are required, redundantly, for lens induction, and demonstrate markedly reduced levels of H3 K18ac and H3 K27ac in the mutated prospective lens ectoderm.

## MATERIALS AND METHODS

### Ethics statement

Animal husbandry and experiments were conducted in accordance with the approved protocol of the Albert Einstein College of Medicine Animal Institute Committee and the ARVO Statement for the use of animals in Ophthalmic and Vision Research.

### Mouse work: crosses

The CBP^flox/+^ (35), p300^flox/+^ (12) and Le-cre (36) lines of mice have been described previously. Cre/GFP expression in Le-Cre mice begins between E8.5 and E9.0, and its expression is restricted to the derivatives of surface ectoderm and is not expressed in the retina (36) (Figure 1). Genotyping of these alleles by polymerase chain reaction (PCR) was conducted with primers described elsewhere (12,36). We also used a second line of mice, termed Pax6-Cre mice, to obtain Cre expression specifically in lens progenitor cells. Unlike the Le-Cre mice, the Pax6-Cre mice do not express Cre in the pancreas. The Pax6-Cre DNA construct was assembled using the following sequences cloned into the Litmus 28 vector: a 1.3 kb HincII to NsiI fragment containing the mouse Pax6 ocular epithelial enhancer from the full-length Pax6 promoter (37) (provided by Dr Peter Gruss, Max-Plack-Institute for Biophysical Chemistry), a 975-bp SalI to BglII fragment containing the mouse P0 Pax6 promoter and transcription initiation site (from −915 to +60), a 1.7-kb EcoRI fragment from pMC1-Cre (38) (obtained from Dr Kirk Thomas, University of Utah) containing the Cre coding sequences followed by neo sequences and an HSV TK polyA sequence, and a 900-bp sequence from SV40 including the small t antigen intron and the early region polyA sequences. This construct does not have the pancreatic enhancer that is present in the Le-Cre mice. The Pax6-Cre transgene (5 kb) (Supplementary Figure S1A) was purified after digestion of the plasmid with SpeI and SacI, then injected into one-cell stage FVB mouse embryos as described (39). Mice were screened by PCR using tail genomic DNA and Cre specific primers, sense: 5′ ATG CTT CTG TCC GTT TGC C 3′, antisense: 5′ CAA CAC CAT TTT TTC TGA CCC 3′, yielding a 650 bp product. In this manuscript, we use the following shorthand nomenclature. Mice with one floxed allele are termed CBP^fl/wt^ or p300^fl/wt^, while mice that are homozygous are CBP^fl/fl^ or p300^fl/fl^. In the presence of a Cre transgene, the floxed alleles are conditionally inactivated in the presumptive lens ectoderm, so we refer to these heterozygous embryos as CBP^wt/−^ or p300^wt/−^ and the homozygotes as CBP^−^^/−^ or p300^−^^/−^. Mice that are both CBP^−^^/−^ and p300^−^^/−^ are termed the double conditional knockout (DCKO) or double mutant mice.

### Hematoxilin and eosin staining, β-galactosidase (X-gal) staining and gross histology

Hematoxilin and eosin staining of paraffin sections was conducted by the Albert Einstein College of Medicine Histotechnology and Comparative Pathology Core Unit. Images were taken with an Axioskop II or AxioObserver Z1 microscope with Axiovision software (Carl Zeiss, Thornwood, NY, USA). Embryos from timed matings of Pax6-Cre mice to ROSA26 reporter mice (abbreviated as R26R) (40), were stained for lacZ activity by incubation with X-gal as previously described (41). After examination and photography, embryos were processed in ethanol and Histoclear (National Diagnostics) and embedded in paraffin for sectioning. Tissue sections were counterstained with Nuclear Fast Red (Poly Scientific).

### Immunofluorescence

Dissected embryos were fixed in 10% formalin or 4% paraformaldehyde overnight. Formalin-fixed embryos were then dehydrated in an ethanol gradient. Tissues were processed, embedded in paraffin and sectioned at 5 µm. Paraffin sections were incubated for 1 h at 60°C, deparaffinized in xylene three times for 5 min, 100% ethanol twice for 3 min, followed by incubation in 95, 80 and 70% ethanol for 1 min each. Antigen retrieval was done by boiling the slides in 10 mM sodium citrate (pH 6.9) for 20 min in a vegetable steamer. Slides were cooled for 20 min and washed twice with phosphate buffered saline (PBS) for 10 min. Embryos fixed in paraformaldehyde were cryoprotected in 30% sucrose, embedded in optimal cutting temperature (OCT) medium and cryosectioned at 7 µm. Slides were washed twice in PBS for 10 min. From here on, both paraffin and frozen sections were processed using the same procedure. Slides were blocked for 30 min at room temperature with Image-iT® FX signal enhancer (Molecular Probes) and washed twice for 10 min in PBS. Slides were incubated with primary antibodies [diluted in PBS containing 1% bovine serum albumin (BSA) and 0.1% Triton-X] overnight at 4°C. Slides were then washed twice in PBS for 10 min and incubated with the secondary antibody (diluted in PBS containing 1% BSA and 0.1% Triton-X) plus 4′,6-diamidino-2-phenylindole (DAPI) (1:50 000, Molecular Probes) for 45 min at room temperature. Sections were washed twice for 10 min in PBS and mounted with VectaShield fluorescent mounting media (Vector).

Primary antibodies used are as follows: Pax6 (1:500, Covance, PRB#278 P); Six3 (1:500, provided by Drs Wei Liu and Guillermo Oliver); Foxe3 [1:500, provided by P. Carlsson (42)]; Sox2 (1:1000, Chemicon, AB5603); αA-crystallin (1:1000, Santa Cruz, sc-22743); αB-crystallin (1:500, Enzo Life Science, ADI-SPA-223); Prox1 (1:2000, Abcam, ab37128); GFP (1:500, Invitrogen, A-11122); H3 K9ac (1:1000, Abcam, ab4441); H3 K18ac (1:100, Abcam, ab1191); H3 K27ac (1:1000, Abcam, ab4729); H3 K4me3 (1:000, Millipore, 07-473); H3 K27me3 (1:1000, Millipore, 07-449); H4 K8ac (1:1000, Abcam, ab15823); H4 K12ac (1:1000, Abcam, ab46983); anti-CBP (1:100, Santa Cruz Biotechnology, A-22); anti-p300 (1:500, Santa Cruz Biotechnology, C-20). Secondary antibodies for immunofluorescence were Alexa Fluor 568 goat anti-rabbit IgG (1: 500, Molecular Probes, A11011) and Alexa Fluor 488 goat anti-rabbit IgG (1:500, Molecular Probes, A11008). Imaging was conducted as previously described (43).

### Quantification of immunofluorescence

The intensities of the immunofluorescence signals of Pax6, Six3, Sox2, H3K9ac, H3K18ac, H3K27ac, H3K4me3 and H3K27me3 in the lens placode and optic vesicle from three sequential slides of each of two embryos from two litters were measured by Image J software from NIH (http://rsbweb.nih.gov/ij/) with a fixed threshold. The tissue areas were outlined in the software program to quantify the total fluorescence within each tissue. The signals were normalized by using the staining in optic vesicle (OV) as an internal control, as in previous published studies from our group (44). Student *t*-tests were performed by R-project tool (http://www.r-project.org/) to establish the significance of changes of the protein levels in wild type (WT) and DCKO mouse PLE.

### BrdU and TUNEL analysis

Proliferation was analyzed by intraperitoneal injection of timed pregnant females with 100 µg BrdU per gram of mouse body weight (45). The females were sacrificed 2 h after injection and embryos were harvested. Yolk sac DNA was used for genotyping. Immunostaining of paraffin sections for BrdU was performed by the Histotechnology and Comparative Pathology Core Unit (AECOM). TUNEL labeling was conducted on paraffin sections using the DeadEnd Fluorometric TUNEL System (Promega). Slides were deparaffinized, rehydrated and TUNEL labeling was performed according to manufacturer’s instructions.

### Laser microdissection of E10.5 mouse embryos and RNA expression profiling

CBP/p300^fl/fl^; Pax6-Cre-positive and -negative E10.5 mouse embryos were genotyped, frozen in OCT medium, sectioned and the lens placode region microdissected using a Leica LMD 6000 laser microdissection system. Total RNAs were reverse transcribed and amplified using the NuGEN Pico system, as described previously (46). Amplified cDNAs from three WT and three double knockout (KO) embryos of each genotype were analyzed on Illumina Mouse6-V2 Bead Chip whole-genome microarrays. Transcripts that were significantly altered in expression in the WT and KO embryos were determined using Illumina GenomeStudio software after cubic spline normalization. The initial data analysis was based on *P* < 0.05 and < 0.01. A list of significantly decreased transcripts (*P* < 0.001 after correction for multiple comparisons) was generated and compared with a list of differentially expressed genes in Pax6 null surface ectoderm (46,47), and to the list of genes with lens-enriched expression in the iSyTE database (48). The NCBI Gene Expression Omnibus reference series for Pax6 and CBP/p300 data sets is GSE49219. The iSyTE accession number is GSE32334.

## RESULTS

### Mice with conditional deletion of CBP or p300 CKO display normal lens morphology

**Figure 2.**
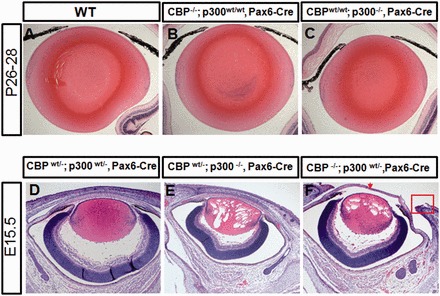
Disrupted lens fiber cell homeostasis following the loss of either combination of three CBP and p300 alleles. (**A**) Normal lens in WT 4-week-old mouse. (**B**) Normal lens appearance of the CBP^−/−^ CKO. (**C**) Normal lens appearance of the p300^−/−^ CKO. (**D**) Normal lens morphogenesis in the double CBP^wt/−^/p300^wt/−^ heterozygous (control) E15.5 embryo. (**E**) Lens fiber cell differentiation in CBP^wt/−^/p300^−/−^ embryo is accompanied by extensive formation of vacuoles. (**F**) In CBP^−/−^/p300wt^/−^ embryos, lens fiber cells also display a similar pattern of vacuolization. In addition, these E15.5 embryos show a defect in eyelid closure (red box) as well as a thinner cornea (red arrow). Pax6-Cre was used for conditional gene inactivation.

Previous KO studies have shown that CBP and p300 null mice are embryonic lethal at approximately E9.0 (7,8). To study the roles of these transcriptional co-activators in lens development, conditional knockout (CKO) CBP and p300 embryos were generated by crossing CBP*^flox^* or p300*^flox^* mice (12) with a novel Pax6-Cre line, which was designed to express Cre recombinase (driven by a Pax6 lens enhancer/promoter) in the lens and corneal precursor cells of the embryonic head ectoderm but not in the pancreas (see Supplementary Figure S1). To verify tissue specificity of the Cre recombinase, we crossed the Pax6-Cre transgenic mice with the Rosa26R LacZ reporter line (40) (see Supplementary Figure S1B). At E10.5, whole mount and tissue sections from bigenic embryos showed LacZ expression specifically in the lens vesicle (see Supplementary Figure S1C and D). By E12.5, we detected LacZ expression throughout the lens (Supplementary Figure S1D), as well as in the corneal (Supplementary Figure S1D), conjunctival and the eyelid epithelial cells, in scattered cells within the retinal neuroectoderm (Supplementary Figure S1D) and in a streak of facial epidermal cells derived from the presumptive ectoderm (Supplementary Figure S1C). Whole mount embryos also exhibited LacZ expression in the forelimbs and digits but not in the pancreas (Supplementary Figure S1C). Mice from Pax6-Cre line OVE1644 were crossed to mice carrying a floxed CBP or floxed p300 allele. Bigenic mice were then inbred to create mice that were Cre positive and homozygous for one of the floxed genes. At postnatal day 26–28, mice with homozygous conditional deletion of CBP or p300 showed no signs of ocular defects when compared with WT mice (Figure 2A–C).

### Compound effects of CBP and p300 inactivation during mouse eye development

The lack of a lens phenotype from the inactivation of CBP or p300, as well as data from previous KO studies, suggested that CBP and p300 are functionally redundant (13). To address this possibility, Pax6-Cre CBP/p300 compound mutant mice were generated. Histological analysis revealed that E15.5 CBP^wt/−^; p300^wt/−^ double heterozygous embryos did not have any distinctive lens defects (Figure 2D). However, E15.5 embryos with conditional inactivation of both alleles of p300 and one allele of CBP showed numerous vacuoles in lens fiber cells (compare Figure 2D and E). Lenses in which CBP was absent and one p300 allele was mutated also displayed lens vacuolization (Figure 2F). In addition, these embryos had a thinner corneal stroma and showed failure of eyelid closure (Figure 2F). These data suggest that lens fiber cells require at least two functional alleles of either CBP or p300, and that one functional allele of p300 cannot fully compensate for loss of CBP during corneal and eyelid development.

### Lens placode morphogenesis is disrupted in CBP/p300 double mutants

**Figure 3.**
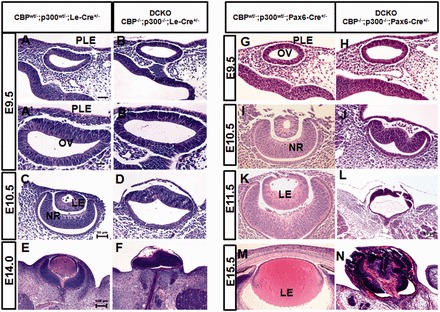
Lenses are not induced in CBP/p300 double mutants (DCKO). (**A–F**) DCKO embryos with the Le-Cre line. At E9.5, the overlying lens ectoderm appears morphologically similar in ‘control’ double heterozygous (A, A’) and double mutant embryos (B, B’). Higher magnifications are shown in panels A’ and B’. At E10.5, the ‘control’ embryo forms a lens vesicle (C). No lens vesicle is formed in the double mutant (D). At E14, the ‘control’ embryo shows a well-developed lens (E). In the CBP/p300 double null mutant, the malformed eye lacks the lens, and retinal and anterior segment development is compromised (F). (**G–N**) DCKO experiments with the Pax6-Cre line. At E9.5, presumptive lens ectoderm appears normal in the double heterozygous ‘control’ (G) and the double mutant embryo (H). At E10.5, the lens vesicle has formed in the ‘control’ embryo (I) but is absent in the double mutant (J). At E11.5, primary lens fiber cell differentiation has initiated in the ‘control’ embryo (K) while no rudimentary lens structure is present in the double mutant (L). At E15.5, the ‘control’ embryo shows a growing lens (M). In the CBP/p300 double null mutant, the malformed eye extrudes from the embryo’s head (N). Abbreviations: PLE, presumptive lens ectoderm; OV, optic vesicle; LE, lens; NR, neural retina.

We next analyzed the phenotypic impact of the loss of both CBP and p300 during lens induction (Figure 3). For this purpose, we used not only the Pax6-Cre mice but also Le-Cre mice where Cre/GFP expression has previously been shown to begin between E8.5 and E9.0 (36) (Figure 1). At E9.0, we did not find any overt differences in the prospective lens ectoderm (head surface ectoderm) between the WT and the double mutant embryos (genotype analyzed: CBP^−^^/−^; p300^−^^/−^, Le-Cre; data not shown). In WT as well as in double heterozygous mouse embryos, the surface ectoderm adjacent to the optic vesicle thickens at E9.5, forming the lens placode (Figure 3A, A’ and G). During this stage, CBP/p300 double mutants formed a structure morphologically similar to the normal lens placode although it was less thickened (Figure 3B, B’ and H). By E10.5, the normal lens placode has invaginated to form a lens pit/early lens vesicle and the neuroepithelium of the optic vesicle has invaginated to form the optic cup (Figure 3C and I). However, the double KO embryos failed to form a lens pit (Figure 3D and J). In addition, the optic vesicle did not invaginate to form the optic cup although the distal optic vesicle was formed (compare Figure 3C and I with 3D and J). At E11.5, the lens vesicle and optic cup have formed in the double heterozygous ‘control’ embryos (Figure 3K). In the mutant embryos, eye development is severely disrupted, with the absence of lens tissue and the presence of a mis-folded retina that protrudes from the head (Figure 3L). At E14.0 and E15.5, while eye development has progressed normally with the formation of differentiating cornea, lens and retina in the double heterozygous ‘control’ embryos, development of the anterior segment and retina in the mutant embryos is severely compromised (compare Figure 3E and F, 3M and N). The defects elicited using Le-Cre for the DCKO of CBP and p300 were comparable with those obtained using Pax6-Cre (Figure 3), implying that the modified Pax6 promoter in the Pax6-Cre mice is active by E8.5–E9. Anophthalmia is visible in DCKO newborn mice (Supplementary Figure S2).

### Depletion of CBP and p300 does not block S phase entry and does not induce apoptosis in the presumptive lens ectodermal cells

**Figure 4.**
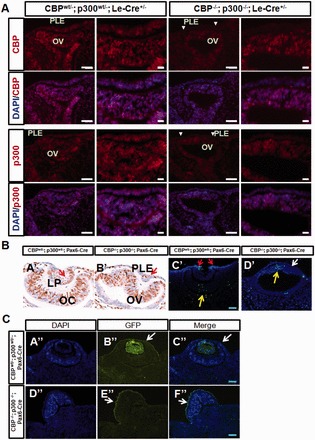
Characterization of CBP/p300 double CKO prospective lens ectoderm. (**A**) Immunostaining for CBP and p300 proteins. Both proteins are depleted in E9.5 mutant (CBP^−/−^; p300^−/−^; Le-Cre^+/−^) presumptive lens ectoderm cells (indicated by white arrowheads) but are detected in the double heterozygous (CBP^wt/−^; p300^wt/−^; Le-Cre^+/−^) ‘control’ ectoderm. The panels on right are higher magnifications of images shown on left. (**B**) Cell proliferation and apoptosis assays in E10.5 embryos. BrdU immunohistochemical staining is shown in the lens pit of a ‘control’ embryo (A’, red arrow). Less BrdU staining is apparent in the ectoderm area of the CBP/p300 DCKO embryo (B’, red arrow). TUNEL labeling is shown in panels C’, D’. Apoptotic cells are not found at the ectoderm of the double mutant (D’, white arrow). In the ‘control’ embryo, apoptosis can be seen in cells (red arrow) at the boundary between the invaginating lens pit and the adjacent surface ectoderm. In both embryos, a cluster of apoptotic cells is seen in the central domain of the retinal neuroblasts (yellow arrows). (**C**) Visualization of GFP expression from the Le-Cre/GFP transgene. GFP expression is driven by the Pax6 promoter in this transgene (36). The GFP staining in the DCKO mutant surface ectoderm at E12.5 (E”, F”, white arrows) provides evidence that the presumptive lens cells are not dying. Moreover, continuous expression of GFP suggests that these cells are not changing their identity after loss of CBP and p300 expression. Panels A”, B” and C”, double heterozygote control; panels D”, E” and F”; DCKO. GFP, green; DAPI, blue. White arrows point to the GFP-positive ectoderm-like cells. Abbreviations: PLE, presumptive lens ectoderm; LP, lens pit; OC, optic cup; OV, optic vesicle. Scale bar = 50 µm.

To confirm that gene targeting of CBP and p300 resulted in depletion of these proteins in the head surface ectoderm, we conducted immunofluorescence analyses using specific antibodies. CBP^wt/−^; p300^wt/−^ embryos showed normal eye formation and were used as ‘controls’ (Figure 4). Both CBP and p300 were found in the lens placode, surface ectoderm, optic cup and periocular mesenchyme (Figure 4A). In contrast, levels of both proteins were substantially reduced in the E9.5 DCKO presumptive lens ectoderm (Figure 4A, indicated by white arrowheads), but not in the optic vesicles (Figure 4A). We next tested whether the aphakic phenotype in the CBP/p300 double mutants could be attributed to defects in cell cycle entry and/or to apoptosis as shown for Six3 mutants (49). BrdU labeling at E10.5 revealed a reduced number of proliferative cells in the double mutant surface ectoderm in comparison with the CBP^wt/−^; p300^wt/−^ (Figure 4B, compare panels A’ and B’), but some cells are still BrdU positive. TUNEL labeling did not show any apoptotic cells within the E10.5 mutant surface ectoderm (Figure 4B, white arrow, compare panels C’ and D’). In addition, ectodermal cells of E12.5 DCKO embryos stained positive for green fluorescent protein (GFP) expression from the Le-Cre transgene (see Figure 4C, panels D” and E”). This provides further evidence that the CBP/p300 double mutated ectoderm-like cells are viable. We conclude that Cre-mediated recombination of the floxed CBP and p300 alleles terminate expression of these enzymes in the surface ectoderm. In addition, our data show that the loss of lens formation is not due to death or disappearance of the lens progenitor cells.

### Lens cell fate specification and determination is altered in CBP/p300 double mutants

**Figure 5.**
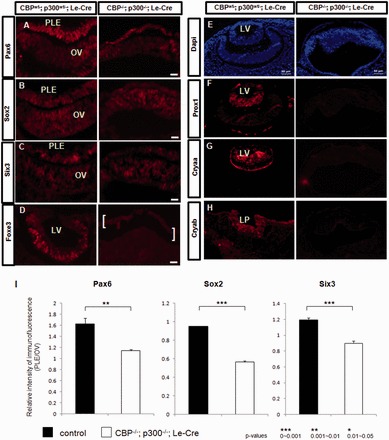
Altered expression of lens cell lineage markers. Immunofluorescent detection of (**A**) Pax6; (**B**) Sox2; (**C**) Six3; (**D**) Foxe3; (**E**) DAPI; (**F**) Prox1; (**G**) αA-crystallin; (**H**) αB-crystallin; and (**I**) Quantitative analysis of Pax6, Sox2 and Six3 expression. In double heterozygous ‘control’ embryos as well as in WT embryos (36,51), expression of Pax6 is upregulated in the presumptive lens ectoderm. In contrast, in DCKO embryos, Pax6 expression is not upregulated (see also quantitative data in Supplementary Figure S3). Expression of Sox2 and Six3 is also reduced in the DCKO PLE (I). FoxE3 expression is readily detected in the cells of the ‘control’ lens vesicle (D), but is not detected in the DCKO PLE in the area between the white brackets (D). Strong Prox1 staining is present in the ‘control’ lens vesicle, but Prox1 is not detected in the DCKO (F). Both αA- and αB-crystallin are expressed in the developing ‘control’ lenses (G, H). In contrast, the mutated prospective lens ectodermal cells do not express these proteins (G, H). Panels (A–C) are E10.5 embryos, panels (E–H) are E11.0–11.5. Abbreviations: LP, lens pit; LV, lens vesicle; PLE, presumptive lens ectoderm; OV, optic vesicle. Scale bar = 50 µm.

Lens progenitor cells are characterized by the expression of the transcription factors Pax6 (36), Six3 (49) and Sox2 (50). Immunostaining for Pax6 in the surface ectoderm at E9.0 did not show any marked difference in expression between CBP^wt/−^; p300^wt/−^ and CBP; p300 null embryos (data not shown). At E9.5, in CBP^wt/−^; p300^wt/−^ embryos, Pax6 is highly expressed throughout the entirety of the invaginating lens placode (Figure 5A). Interestingly, in the mutant ectoderm, Pax6 expression was retained, but the signal was reduced (Figure 5A). Reduced amounts of Six3 and Sox2 were also found in the mutated surface ectoderm compared with the CBP^wt/−^; p300^wt/−^ invaginating lens placode (Figure 5B and C). Staining intensities for Pax6, Six3 and Sox2 were quantified and the results are shown in Figure 5I and Supplementary Figure S3. Expression of Pax6 was also evaluated at E10.5 and E11.5 stages. The results show persistently reduced expression of Pax6 in DCKO mutants (Supplementary Figure S3). Foxe3, a lens-specific marker of lens induction (42), is expressed in CBP^wt/−^; p300^wt/−^ embryos in the invaginating lens pit (Figure 5D). In CBP/p300 DCKO mutants, expression of Foxe3 was undetected in the presumptive lens ectoderm (Figure 5D). Loss of Foxe3 induction is consistent with the aborted lens formation in the CBP/p300 double mutants. Prox1 is a DNA-binding transcription factor required for expression of γ- (52) and β-crystallins (53,54). Immunofluorescence staining for Prox1 in E11–E11.5 CBP^wt/−^; p300^wt/−^embryos showed Prox1 positive cells predominantly in the posterior part of the lens vesicle as previously reported (55) (Figure 5F). No Prox1 staining was detected in the CBP/p300 double mutant embryos (Figure 5F). Expression of crystallins has been proposed to define the ‘determined state’ for lens progenitor cells (Figure 1) (15). Immunofluorescence staining for αA- and αB-crystallins was conducted in both CBP^wt/−^; p300^wt/−^and DCKO mutant embryos. Expression of both crystallins was confirmed in CBP^wt/−^; p300^wt/−^ embryos, but neither protein was detected in mutant ectoderm (Figure 5G and H). Thus, lens cell determination, i.e. the formation of committed lens progenitor cells marked by the expression of α-crystallins, was not achieved in the CBP/p300 double mutants.

To gain additional insights into the status of the presumptive ectoderm, we examined expression of p63, E-cadherin and α-actin. Transformation-related protein 63 (p63), a transcription factor from the p53/p63/p73 gene family (56), is proposed to function in the commitment, development and maintenance of epithelial cells (57,58). This factor also serves as a marker for highly proliferative stem-like cells (59,60). In CBP^wt/−^; p300^wt/−^ embryos, we found that p63 was expressed in the surface ectoderm of the presumptive cornea and within the lens vesicle cells attached to this surface ectoderm, but p63 expression was silenced in the cells that had invaginated to form the lens pit. In contrast, in CBP/p300 mutants, we found complete loss of p63 expression within this region (Supplementary Figure S4, compare panels A and B), implying that p63 expression requires CBP/p300 or that p63 silencing does not require histone acetylation. Cadherins are cell adhesion molecules, which have been shown to be important in lens vesicle separation, lens epithelial cell adhesion and survival (61). During lens development, N-cad, P-cad and E-cad have distinct spatial and temporal patterns of expression (62). Cadherins form protein complexes with catenins, and these complexes associate with F-actin. We analyzed both F-actin and E-cadherin in the E11.0–E11.5 CBP/p300 double mutants. Expression of E-cadherin does not change between the ‘control’ CBP^wt/−^; p300^wt/−^ and DCKO mutant embryos (Supplementary Figure S4, compare panels C and D). Based on phalloidin staining, F-actin in the double heterozygous lens precursor cells exhibited the typical pattern of higher apical localization (Supplementary Figure S4E, G). F-actin in the mutant embryo was more symmetric, with strong staining both apically and basally in some cells (Supplementary Figure S4, compare panels G and H).

### Identification of differentially expressed genes in lens placode/surface ectoderm between WT and CBP/p300 DCKO embryos

To identify genes whose expression is altered upon loss of CBP and p300 during lens placode formation, we performed whole-genome RNA expression profiling using bead microarrays (Illumina) to evaluate the transcriptome of the mutated surface ectoderm. We compared gene expression levels in E10.5 WT and CBP/p300 DCKO head surface ectoderms. The RNAs were isolated following laser microdissection as described in ‘Materials and Methods’ section. Three sets of biological replicates (3 WT and 3 KO) were analyzed by Illumina MouseWG-6 v2.0 Expression BeadChip. Statistical and bioinformatics analyses were performed as described in ‘Materials and Methods’ section. In the CBP/p300 null embryos, we initially identified 2418 transcripts that were significantly differentially expressed when compared with the WT head surface ectoderm/lens placode. Among them, 1569 were significantly decreased and 849 were increased in expression (*P* < 0.05). Selected differentially expressed genes known to be relevant to lens morphogenesis or lens function (*P* < 0.001) are shown in Table 1. Notably, expression of a number of crystallin genes, including Crybb3, Cryge, Crygd and Cryba2, was reduced >100-fold. We next compared these results with genes that were differentially expressed in Pax6 null ectoderm (46,47). A total of 231 transcripts were decreased in expression in both the Pax6 and CBP/p300 CKOs (*P* < 0.05), including the DNA-binding transcription factors MafB, c-Maf, Sox2, Otx1, Prox1, Pitx3, Foxe3 and Zeb2. These data show that the disrupted lens placode formation in both Pax6 and CBP/p300 mutants is caused through shared molecular mechanisms that converge on a set of genes critical for lens lineage formation, and for subsequent stages of lens development. Among the genes that were decreased in CBP/p300, but not in Pax6 CKOs, previous genetic studies demonstrated that specific genes, including BMP7 (85), Cited2 (81), Bcor (86), Sox11 (87,88) and N-Myc (89), play unique roles in lens development. Transcripts that showed the most statistically significant decreases in expression both in the absence of Pax6 and in the absence of CBP/p300 are highlighted in Supplementary Table S1.

**Table 1.** Transcripts with known function in the lens that decreased significantly in CBP/p300 knockout E10.5 lens placodes (listed in order of decreasing statistical significance)

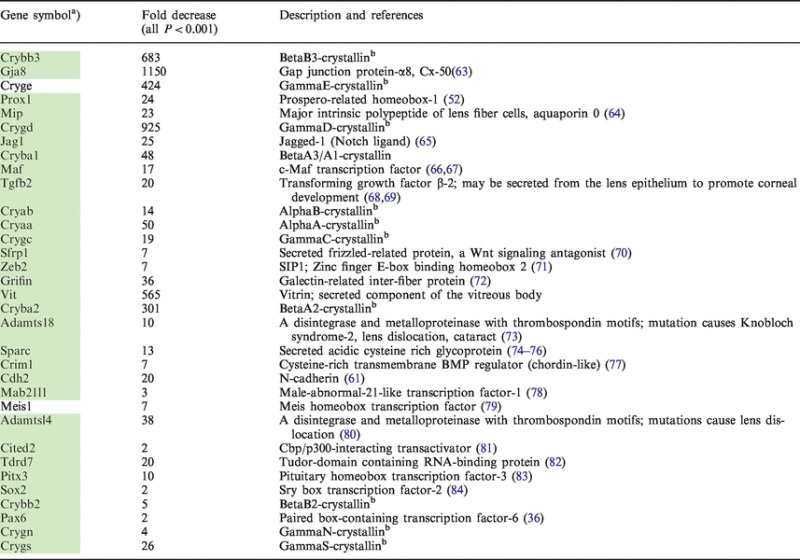

^a^Genes shown in green field can be also found in the iSyTE.

^b^The primary function of the crystallin genes is assumed to be their contribution to the transparency and refractive power of the lens.

We also compared the transcripts that were decreased in the CBP/p300 mutant ectoderm to genes in the iSyTE database. The iSyTE database contains transcripts that are preferentially expressed in the developing lens, compared with the whole embryo (48). Of the top 563 transcripts that were significantly decreased in expression in the CBP/p300 mutants (*P* < 0.01), 205 (36%) were preferentially expressed in the lens either at E10.5, E11.5 or E12.5 (*P* < 0.004) (Supplementary Table S1). Notably, the top 50 differentially expressed genes in CBP/p300 mutants include 34 (68%) genes identified by the iSyTE analysis (Supplementary Table S1). Overall, the transcripts that are dependent for their expression on CBP/p300 at E10.5 include both ‘lens-enriched’ transcripts and transcripts that are expressed in other cell types during embryogenesis.

### Disrupted histone acetylation patterns in the mutated ectoderms

**Figure 6.**
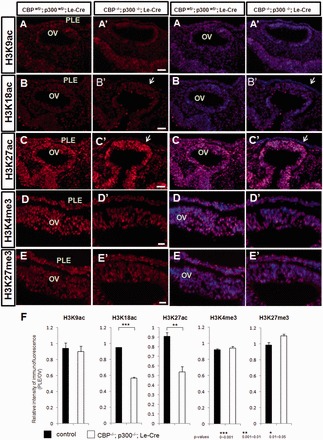
Patterns of histone acetylation and methylation. Immunofluorescence, two left columns; immunofluorescence/DAPI, two right columns. Detection of H3 K9ac in control (**A**) and mutated embryos (A’). Detection of H3 K18ac in control (**B**) and mutated embryos (B’). Detection of H3 K27ac in control (**C**) and mutated (C’) embryos. Detection of H3 K4me3 in control (**D**) and mutated (D’) embryos. Detection of H3 K27me3 in control (**E**) and mutated (E’) embryos. Quantitative analysis of immunofluorescence of H3K9ac, K18ac, K27ac, K4me3 and K27me3 (**F**). Levels of H3 K18ac and H3 K27ac are markedly reduced (white arrows) in the presumptive lens ectodermal cells of the double CKO embryos. The presumptive lens ectoderm showing normal levels of histone PTMs is labeled by yellow arrow. Quantitative analyses of histone acetylation in presumptive lens ectoderm (PLE) are shown in (F). Abbreviations: PLE, presumptive lens ectoderm; OV, optic vesicle.

Finally, to examine histone PTMs during normal and aborted lens placode formation, we used antibodies against specific acetylated and methylated histone H3 proteins. Levels of H3 K9ac, a modification known to be catalyzed by HATs other than the CBP/p300 KAT3 subfamily (1), were not significantly altered (Figure 6A, A’). In contrast, in the double mutant presumptive lens ectoderm, H3 K18ac and H3 K27ac were markedly reduced (Figure 6B, B’ and C, C’) as further shown by quantitative analysis of the immunofluorescence data (Figure 6F). We also examined H4 K8ac and K12ac PTMs. In E9.5 embryos, the levels of these modifications were low compared with E12.5 mouse embryos (Supplementary Figure S5). No changes of H4 K8ac and H4 K12ac were found between the E9.5 control and DCKO embryos (Supplementary Figure S5). Interestingly, acetylation of H4 in the E12.5 lenses seemed to be increased during the initial stages of fiber cell formation (Supplementary Figure S5C’ and F’). We next assayed for changes in the methylation status of histone H3. Although we found no changes using antibodies that recognize H3 K4me3, we did see a subtle increase in H3 K27me3 (Figure 6D–F). Collectively, our data show that inactivation of CBP/p300 results in reduced expression of both proteins followed by selective reduction in acetylated H3 K18 and K27 in the mutated head ectoderm. Consequently, the lens progenitor cells attenuate expression of many transcripts encoding proteins critical for lens development and lens morphogenesis. The processes of lens induction and lens cell specification progress no further.

## DISCUSSION

The present data demonstrate that CBP and p300 are essential genes for lens induction. The data suggest that KAT3 HAT activity, mediated by CBP and p300 is required in the Pax6-expressing prospective lens cells for lens induction. Almost all molecular and morphological aspects of lens placode development (Figure 1) are severely disrupted within ∼12 h after the targeted inactivation of both CBP and p300 by Le-Cre. This result supports the notion that the presumptive competent lens precursor cells are uncommitted and developmentally flexible before the arrival of the optic vesicle (24–26). The absence of expression of Foxe3, Prox1 and αA- and αB-crystallins provides strong evidence that lens determination (Figure 1) is not achieved.

In addition, our results suggest that acetylation of core histones, and possibly other substrates, plays a central role in the process of commitment to the lens differentiation program. Although 18 additional KATs are found in mouse genome, they do not compensate for the loss of CBP and p300. The discontinuation of lens cell determination occurred concomitant with a significant reduction in global acetylation of H3 K18 and K27 residues in the prospective lens ectoderm (Figure 6).

In the double CBP/p300 mutant embryos, the aborted state of lens development phenocopies the defects that are seen following conditional inactivation of the mouse Pax6 gene using the Le-Cre driver (33,36). The previous detection of a Pax6-p300 complex, shown in nuclear extracts from pancreatic β-cells (90), suggests that CBP/p300 and Pax6 may be coordinately recruited to a common set of target genes (91). At the molecular level, RNA expression profiling revealed that 231 (14.7%) of 1569 differentially downregulated transcripts are shared between the CBP/p300 and Pax6 conditional gene targeting models. This difference between genes downstream of Pax6 and CBP/p300 may reflect the fact that CBP/p300 can be targeted to other sites in the genome by other transcription factors in addition to Pax6 (90), that Pax6 may interact with other co-activators at certain target sites in the genome, and/or the fact that the CBP/p300 double mutants continue to express Pax6 in the mutated ectoderm. Although Pax6 expression is moderately reduced in the CBP/p300 double mutant ectoderm, this would not explain the defects in lens placode formation as inactivation of one Pax6 allele delays lens placode formation but does not eliminate lens formation (51). The reduced expression of many genes that are critical for lens formation, including c-Maf, Foxe3, Otx1, Pitx3, Prox1, Six3, Sox2 and Zeb2, supports the idea that depletion of CBP and p300 disrupts the ‘core’ Pax6-dependent genetic machinery of lens cell fate determination and differentiation (Six3 ⇆ Pax6 ⇆ Sox2). Many of the downregulated genes are included in the iSyTE database of genes (48) (Supplementary Table S1), a database that contains a highly enriched population of lens disease genes and serves as a unique resource for novel gene discoveries (82). Taken together with cataracts found in many patients with CBP mutations (10,11), we propose that the genes that are downregulated in the CBP/p300 mutants provide an additional source of candidate genes for human cataracts.

The CBP/p300-regulated group of genes includes BMP7 (85,92) (not in Supplementary Table S1, *P* < 0.01, but in the 1569 gene list, *P* < 0.05). Although BMP4 and BMP7 are growth factors required for lens progenitor cell formation (85,92–94), the CBP/p300-dependent expression of BMP7 in the lens placode could be important for autocrine signaling within the surface ectoderm. In addition, lens morphogenesis, but not lens induction, requires the FGF receptors (95,96). The nuclear targets of FGF signaling in the lens are poorly characterized. Nevertheless, studies of FGF signaling in other systems have shown key roles for AP-1 and Ets transcription factors (97,98). Reduced expression of c-Jun, an AP-1 factor and Etv5/ERM, an Ets factor, is found in the CBP/p300-depleted PLE. Interestingly, both c-Jun and Etv5/ERM are expressed in the embryonic lens vesicle in temporal/spatial patterns, suggesting they may play a role in lens differentiation (Q.X. and A.C., unpublished data).

Our data demonstrate that commitment to the lens cell differentiation program does not occur in the absence of CBP and p300. Although acetylation of H3 K18 and K27 can be catalyzed *in vitro* by other enzymes, such as PCAF, Tip60 and SRC-1, our results show that CBP and p300 catalyze these modifications in the cells of the mouse lens placode. Low abundance of H4 K8ac and H4 K12ac in the presumptive lens ectoderm of E9.5 mouse embryos suggests that these H4 modifications are not required for lens induction. The presence and distribution of other acetylated versions of the histones, such as H3 K14ac and H3 K23ac, H2A K5ac, H2B K12ac and K15ac, and H4 K5ac, remain to be determined in the lens placode. It has previously been proposed that histone PTMs are needed to establish cell type memory during embryonic development (31,99,100). Our results provide *in vivo* support for this prediction because we show that CBP and p300 are necessary for the coordinated embryonic determination of cellular identity for all of the cells that normally form the lens. Another possibility is that the mutant lens cells can no longer acetylate certain critical transcription factors that are expressed in the preplacodal surface ectoderm including Etv1/ER81 (101), Ets1 (102), GABPα (103), CtBP2 (104) and/or β-catenin (105). Interestingly, β-catenin inhibits lens formation and its activity is blocked in the surface ectoderm (106). These two modes of CBP/p300 action are not mutually exclusive.

Abnormal lens fiber cell differentiation in mouse embryos with one remaining functional allele of either CBP or p300 is in agreement with earlier studies of these proteins in the lens. The function of CBP and p300 in lens development was first probed using lens-specific expression of a T-antigen mutant (E107KΔ) in transgenic mice (107). This truncated protein, which was known to associate with CBP/p300 and to interfere with their functions, was found to severely impair the lens fiber cell differentiation program (107,108). Additional studies indicated a direct interaction of CBP/p300 with c-Maf (109), a regulator of crystallin gene expression (66,67,110–114). Co-transfection of c-Maf and CBP/p300 activated the αA-crystallin promoter in cell culture reporter assays (109). Expression of CBP is upregulated in differentiating lens fibers (115). In contrast, expression levels of p300 are similar in both lens epithelium and lens fibers (115). Chromatin immunoprecipitation studies previously identified both CBP and p300 at the mouse αA-crystallin locus (115). Finally, a study of patients with Rubinstein–Taybi syndrome caused by mutations in CBP found 15 congenital cataracts in 81 case reports (11).

Our CBP and p300 gene dosage studies suggest that, in the absence of p300, a single functional allele of CBP is sufficient for normal corneal development and eyelid closure, but in the absence of CBP, a single functional allele of p300 is no longer sufficient for these developmental pathways. It is noteworthy that expression of p300 is paradoxically higher than CBP in the cornea and eyelid (115). In addition, a single residual allele of either CBP or p300 is sufficient for lens formation but insufficient to allow lens fiber cells to maintain their normal phenotype.

In summary, CBP and p300 are required redundantly for successful progression down the pathway of lens differentiation. Our data indicate that they are required for the conversion of competent lens progenitor cells to determined (committed) lens cells, a process that is relatively irreversible. CBP and p300 proteins coordinately regulate the expression of a large cohort of critical genes, and they are important enzymes that determine the levels of acetylated K18 and K27 residues in histone H3 tails in the lens precursor cells. These enzymes may help to establish an epigenetic signature on the genes that are central to the lens differentiation program. We propose that lens cell fate may be achieved in part through the establishment of the requisite histone code (100,116,117) on a widespread cohort of genes that are essential for this unique differentiation program. The location/map (or plat) of these histone modifications, which could be referred to as a histoplat, may establish or dictate cellular identity and may represent a critical aspect of the partitioning system that is implemented to allow a single genome to be reconfigured as necessary to provide the distinct gene expression patterns that are needed to specify a collection of distinct embryonic and adult cell types.

## SUPPLEMENTARY DATA

Supplementary Data are available at NAR Online.

## FUNDING

NIH [EY014237 to A.C., EY014237S1 to A.C., EY04853 to D.C.B., EY021505 to D.C.B. and S.A.L.]; Ocular Development Fund (PAO); unrestricted departmental grants from Research to Prevent Blindness to the Departments of Ophthalmology and Visual Sciences at the Albert Einstein College of Medicine and Washington University School of Medicine; Microarray analysis by the Genome Technology Access Center at Washington University was supported in part by a grant to the Institute of Clinical and Translational Sciences [UL1 TR000448]; Irma T. Hirschl Career Scientist Award (to A.C.). Funding for open access charge: NIH [EY014237 to A.C.].

*Conflict of interest statement*. None declared.

## Supplementary Material

Supplementary Data
