# Supplementary material for: Histone posttranslational modifications and cell fate determination: lens induction requires the lysine acetyltransferases CBP and p300
Source: Nucleic Acids Res. 2013 Sep 12;41(22):10199–214. doi: 10.1093/nar/gkt824 (PMC3905850; doi:10.1093/nar/gkt824)
Supplement: Supplementary Data [file supp_41_22_10199__index.html]

Histone posttranslational modifications and cell fate determination: lens induction requires the lysine acetyltransferases CBP and p300 — Histone posttranslational modifications and cell fate determination: lens induction requires the lysine acetyltransferases CBP and p300 — Supplementary Data 

# Histone posttranslational modifications and cell fate determination: lens induction requires the lysine acetyltransferases CBP and p300

## Supplementary Data

files

**Files in this Data Supplement:**

- Supplementary Data - pdf file
- Supplementary Data - xlsx file
