# Supplementary material for: Histone posttranslational modifications and cell fate determination: lens induction requires the lysine acetyltransferases CBP and p300
Source: Nucleic Acids Res. 2013 Sep 12;41(22):10199–214. doi: 10.1093/nar/gkt824 (PMC3905850; doi:10.1093/nar/gkt824)
Supplement: Supplementary Data [file supp_gkt824_nar-01527-v-2013-File009.pdf]

Supplementary Figure S1

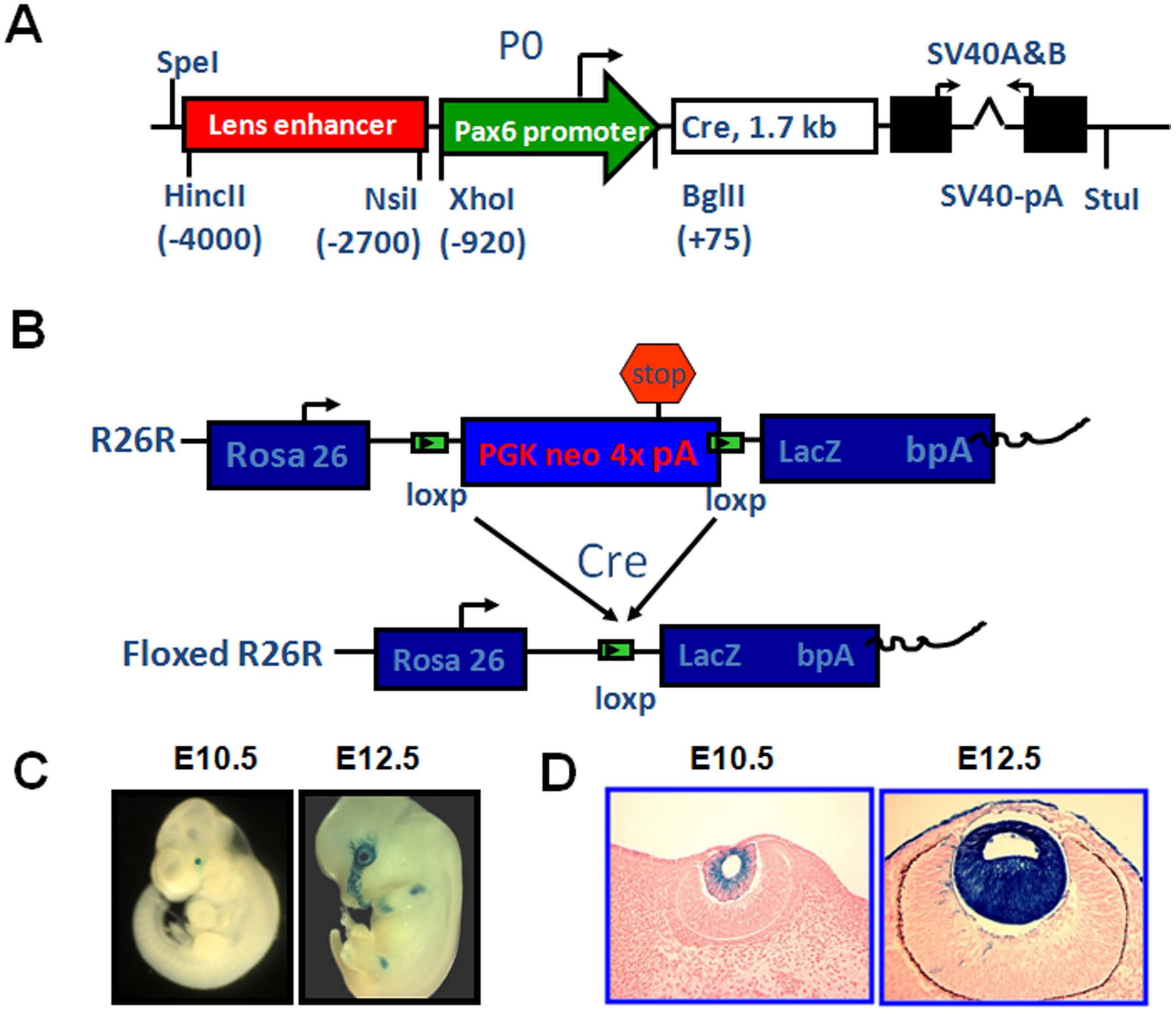

Supplementary Figure S2

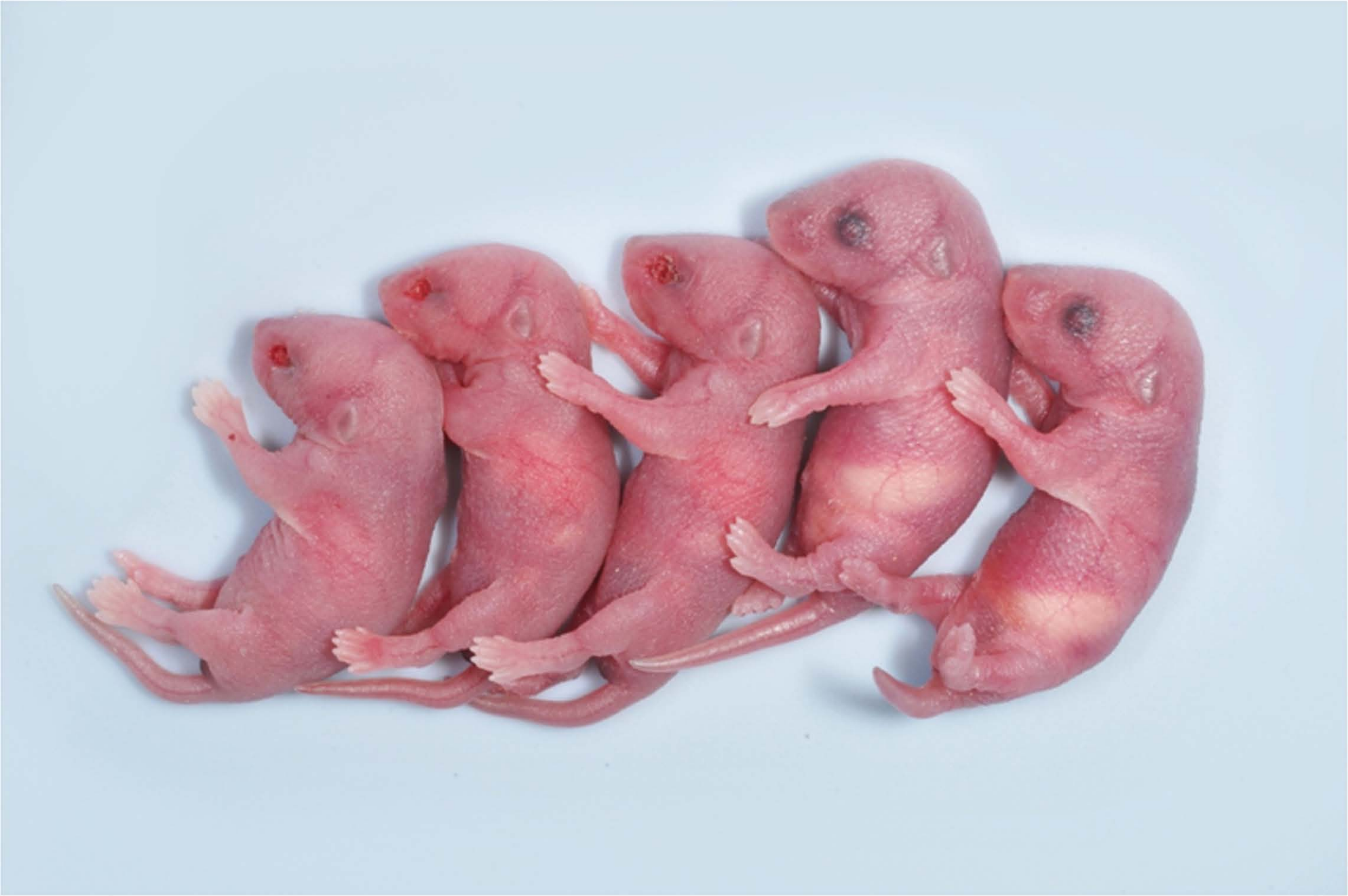

Supplementary Figure. S3

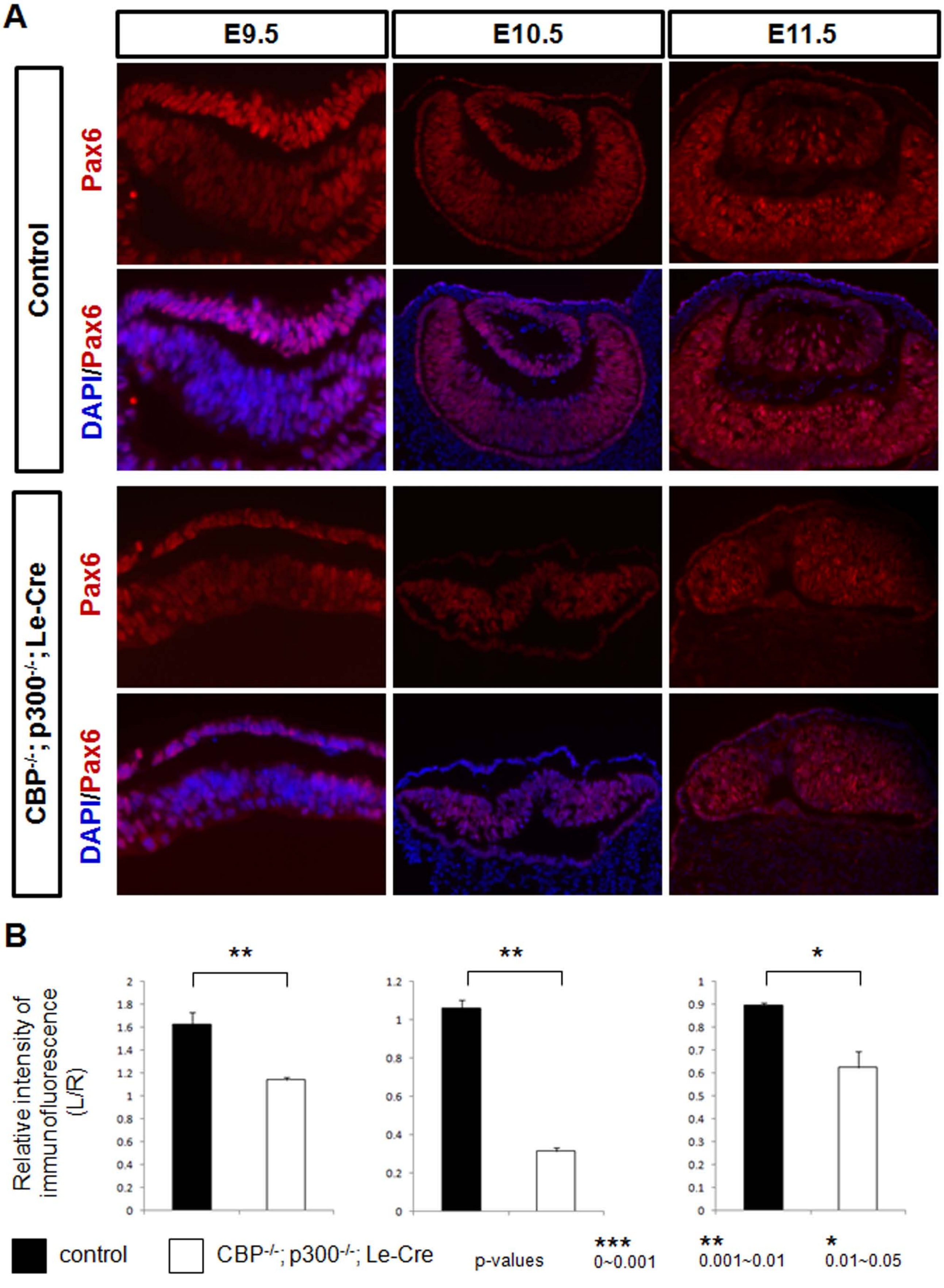

Supplementary Figure S4

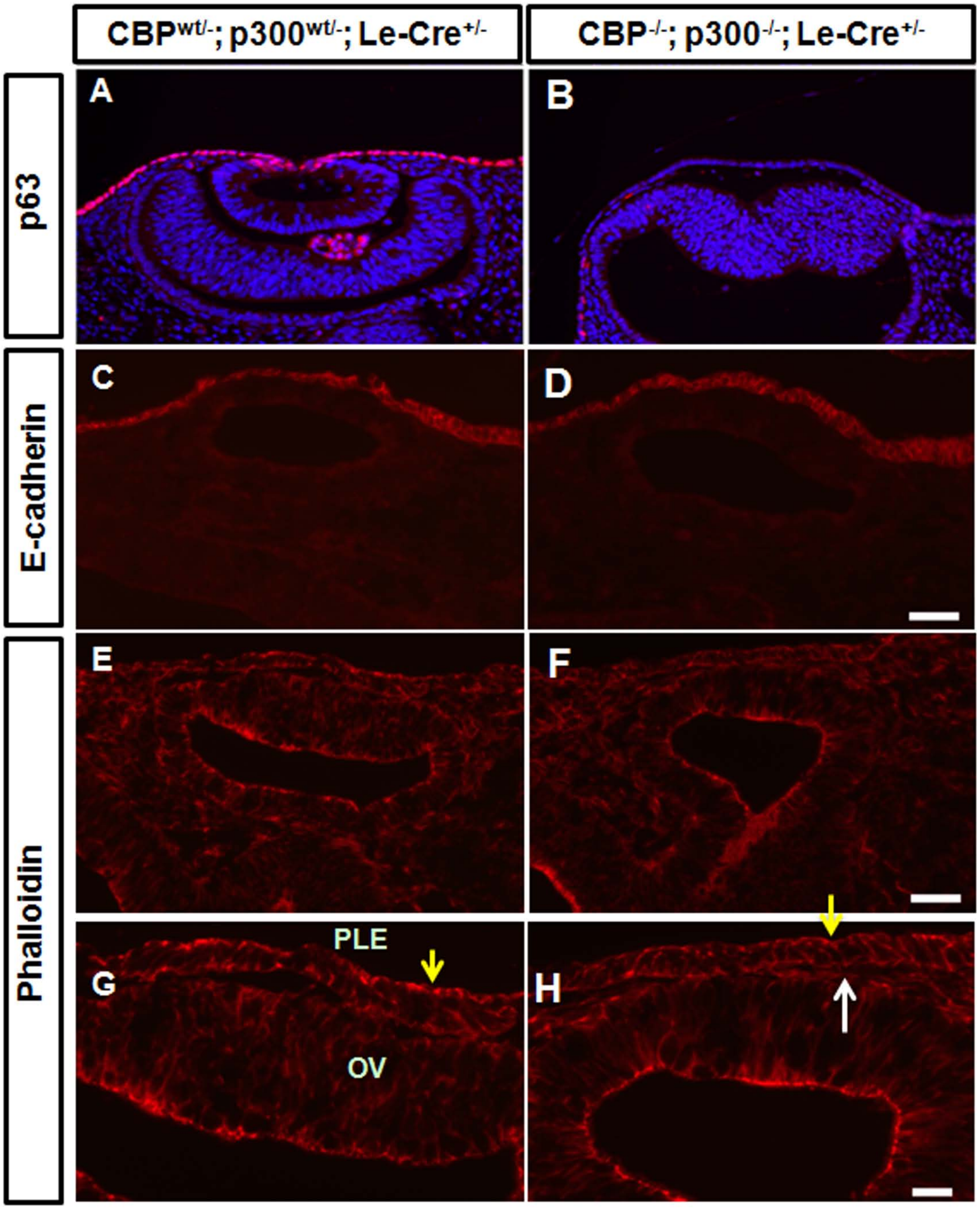

Supplementary Figure S5

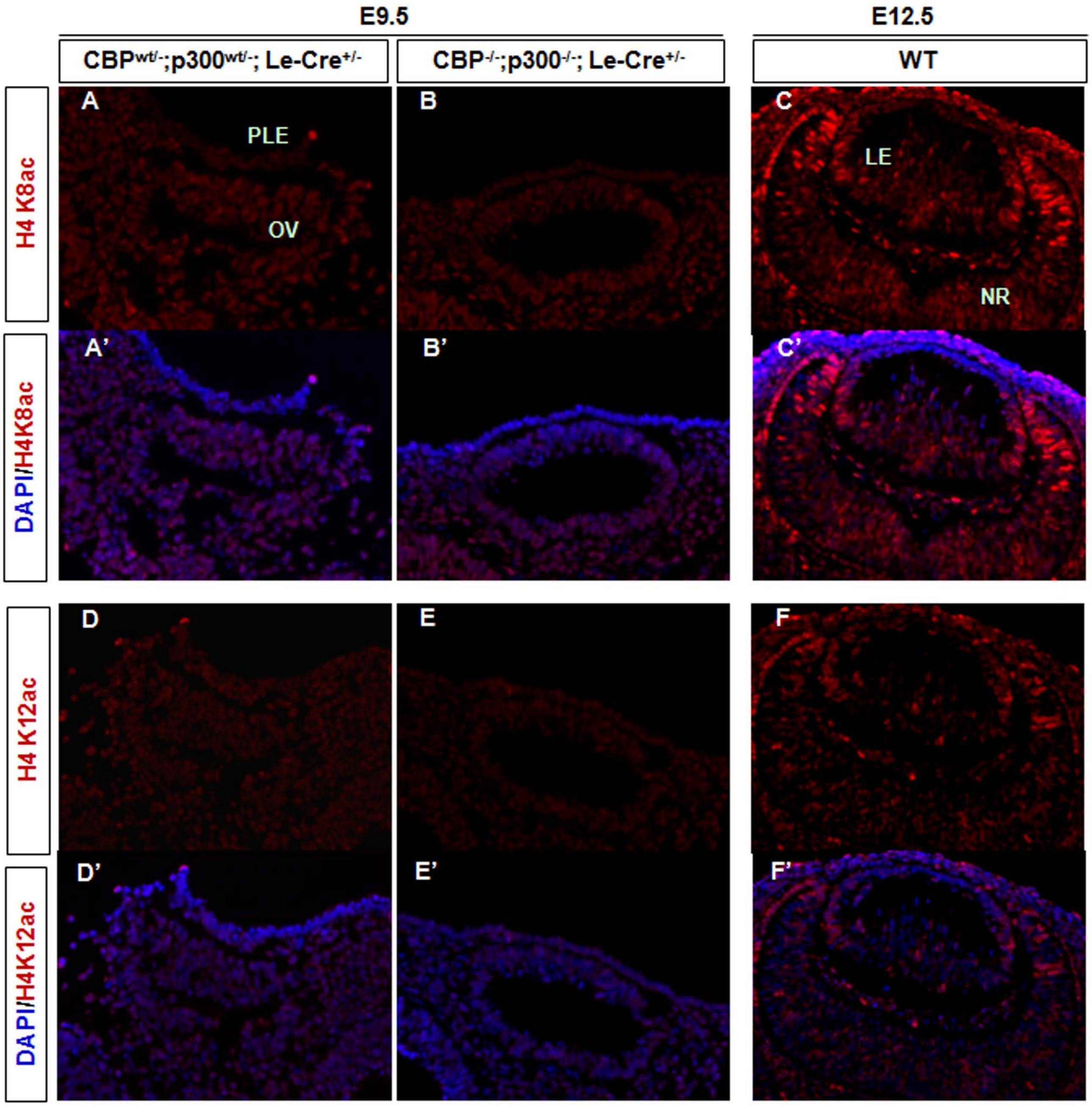

SUPPLEMENTAL DATA

Supplementary Table S1 (.xls)

Supplementary Figure S1: Pax6-Cre transgene. (A,B) Schematic diagrams of the Pax6-Cre construct (A) and the Rosa26R (R26R) allele. Cre expression in mice that are bigenic for Pax6-Cre and R26R will result in excision of the floxed PGK-neo-STOP cassette, and subsequent expression of the LacZ reporter (B). (C,D) LacZ staining in E10.5 and E12.5 embryos. At E10.5, whole mounts (C) and tissue sections (D) show that Cre activity is limited to the lens vesicle. At E12.5, X-gal staining is seen in all cells of the lens (D), all cells of the corneal ectoderm (D), a few scattered cells in the neural retina (D), a streak of ectodermal cells that runs from the eye field to the whisker field in the face (C) and a few distinct clusters of cells in the forelimbs and hindlimbs (C).

Supplementary Figure S2: Anophthalmia of newborn DCKO mice. Pax6-cre was used. The 3 newborns on the left are DCKO and have anophthalmia accompanied by an opening in the surface ectoderm in the location where the eye would normally be. A few pigmented cells can be detected under the skin in the region of the eye primordium in the DCKO newborns. The 2 newborns on the right are negative for Cre.

Supplementary Figure S3: Expression of Pax6 in “control” double heterozygous and DCKO E9.5, E10.5 and E11.5 embryos. A) Immunofluorescence detection of Pax6. B) Quantitative analysis of Pax6 expression.

Supplementary Figure S4: Investigation of epithelial cell markers: transformation related protein 63 (p63), E-cadherin and F-actin in DCKO mice. p63 (A-B), E-cadherin (C-D), and phalloidin staining (E-H) are shown. Panels (G) and (H) are higher magnifications of (E) and (F), respectively. Expression of p63 was evaluated by *in situ* hybridization. In the double heterozygous “control” embryo, p63 is expressed in the head surface ectoderm, but is silenced in the invaginated cells of the lens vesicle (A). In the DCKO embryo, the PLE cells have downregulated p63 expression (B). E-cadherin expression is unaltered (C, D). Apical and basal locations of F-actin are labeled by yellow and white arrows, respectively. Abbreviations: PLE, presumptive lens ectoderm; OV, optic vesicle, OV. Scale bar = 20 μm.

Supplementary Figure S5: Detection of acetylated H4 K8 and H4 K12. Immunostaining for H4 K8ac in “control” double heterozygous E9.5 (A, A’), DCKO mutated E9.5 (B, B’), and E12.5 wild type (C, C’) embryos. Immunostaining for H4 K12ac in “control” double heterozygous E9.5 (D, D’), DCKO mutated E9.5 (E, E’), and E12.5 wild type (F, F’) embryos. Abbreviations: PLE, presumptive lens ectoderm; OV, optic vesicle; LE, lens; NR, neural retina; wild type, WT.
